# Supplementary material for: The Mediating Effects of Chronic Diseases in the Relationship Between Adverse Childhood Experiences and Trajectories of Depressive Symptoms in Later Life: A Nationwide Longitudinal Study
Source: Healthcare (Basel). 2024 Dec 16;12(24):2539. doi: 10.3390/healthcare12242539 (PMC11675985; doi:10.3390/healthcare12242539)
Supplement: Supplementary file 1 [file healthcare-12-02539-s001.zip › healthcare-3343387-supplementary.pdf]

Supplementary materials

Table S1. Items for calculating CES-D-10.

Items	Definition
1. Bothered by little things	Rarely or none of the time < 1 day=0<br>Some or a little of the time 1-2 days=1<br>Occasionally or a moderate amount of 3 days=2<br>Most or all of the time 5-7 days=3
2. Had trouble keeping my mind on what I was doing	Rarely or none of the time < 1 day=0<br>Some or a little of the time 1-2 days=1<br>Occasionally or a moderate amount of 3 days=2<br>Most or all of the time 5-7 days=3
3. Felt depressed	Rarely or none of the time < 1 day=0<br>Some or a little of the time 1-2 days=1<br>Occasionally or a moderate amount of 3 days=2<br>Most or all of the time 5-7 days=3
4. Felt that everything I did was an effort	Rarely or none of the time < 1 day=0<br>Some or a little of the time 1-2 days=1<br>Occasionally or a moderate amount of 3 days=2<br>Most or all of the time 5-7 days=3
5. Felt hopeful about the future	Rarely or none of the time < 1 day=3<br>Some or a little of the time 1-2 days=2<br>Occasionally or a moderate amount of 3 days=1<br>Most or all of the time 5-7 days=0
6. Felt fearful	Rarely or none of the time < 1 day=0<br>Some or a little of the time 1-2 days=1<br>Occasionally or a moderate amount of 3 days=2<br>Most or all of the time 5-7 days=3
7. Sleep was restless	Rarely or none of the time < 1 day=0<br>Some or a little of the time 1-2 days=1<br>Occasionally or a moderate amount of 3 days=2<br>Most or all of the time 5-7 days=3
8.Was happy	Rarely or none of the time < 1 day=3<br>Some or a little of the time 1-2 days=2<br>Occasionally or a moderate amount of 3 days=1<br>Most or all of the time 5-7 days=0
9. Felt lonely	Rarely or none of the time < 1 day=0<br>Some or a little of the time 1-2 days=1<br>Occasionally or a moderate amount of 3 days=2<br>Most or all of the time 5-7 days=3
10. Could Not Get Going	Rarely or none of the time < 1 day=0<br>Some or a little of the time 1-2 days=1<br>Occasionally or a moderate amount of 3 days=2<br>Most or all of the time 5-7 days=3

Notes: CES-D-10, the ten-item Center for Epidemiological Studies Depression Scale.

Table S2. Detailed questions and measurements of ten items ACE scale.

Items	Description of questions	Definition
1.Physical abuse	When you were growing up, did your female/ male guardian ever hit you?	often/sometimes=1<br>rarely/never=0
2.Emotional Neglect	How much love and affection did your female guardian give you while you were growing up?	rarely/never=1<br>often/sometimes=0
	How much effort did your female guardian put into watching over you?	a little/not at all=1<br>a lot/some=0
3.Household substance abuse	During the years you were growing up, did your female/male guardian ever have alcoholism or drug?	yes=1<br>no=0
4.Household mental illness	Did your female/male guardian have abnormality of mind when you were young?	yes=1<br>no=0
	During the years you were growing up, had your female/male guardian showed continued signs of sadness or depression that lasted two weeks or more?	during all/most/=1<br>some/only a little of the childhood=0
5.Domestic violence	Have your father/mother ever beat up your mother/father / Quarrel?	often/sometimes=1<br>not very often/never=0
6.Unsafe neighborhood	Was it safe being out alone at night in the neighborhood where you lived as a child?	not very safe/not safe at all=1<br>very safe/somewhat safe=0
7.Bullying	When you were a child, how often were you picked on or bullied by kids in your neighborhood?	often/sometimes=1<br>not very often/never=0
	When you were a child, how often were you picked on or bullied by kids in your school?	often/sometimes=1<br>not very often/never=0
8.Parental death	Either of the parents was dead before participant was 17 years.	yes=1<br>no=0
9.Incarcerated household member	During the years you were growing up, have your female/male guardian ever been arrested or sent to prison?	yes=1<br>no=0
10.Parental separation or divorce	Were your biological parents divorced (including long separation due to emotional problems) before you were 17 years?	yes=1<br>no=0

Notes: ACEs, adverse childhood experiences.

Table S3. Baseline characteristics of participants with different ACEs scores.

Variables	Total (N = 6921)	ACEs scores 0 (N = 2119)	1 (N = 2365)	2 (N = 1433)	3 (N = 673)	≥4 (N = 331)	*p* Value
Age, years	57.2 ± 8.0	56.8 ± 7.8	57.4 ± 8.1	57.1 ± 8.2	58.1 ± 8.1	57.7 ± 8.3	<0.005
Gender, N (%)							<0.001
Male	3188(46.1)	887(41.9)	1103(46.6)	692(48.3)	351(52.2)	155(46.8)	
Female	3733(53.9)	1232(58.1)	1262(53.4)	741(51.7)	322(47.8)	176(53.2)	
Marital status, N (%)							0.440
Married/cohabiting	6284(90.8)	1938(91.5)	2138(90.4)	1301(90.8)	614(91.2)	293(88.5)	
Unmarried/separated	637(9.2)	181(8.5)	227(9.6)	132(9.2)	59(8.8)	38(11.5)	
Hukou status, N (%)							0.202
Agricultural hukou	5702(82.4)	1724(81.4)	1935(81.8)	1196(83.5)	569(84.5)	278(84.0)	
Non-agricultural hukou	1219(17.6)	395(18.6)	430(18.2)	237(16.5)	104(15.5)	53(16.0)	
Current residence, N (%)							0.513
Urban	2378(34.4)	732(34.5)	836(35.3)	484(33.8)	214(31.8)	112(33.8)	
Rural	4543(65.6)	1387(65.5)	1529(64.7)	949(66.2)	459(68.2)	219(66.2)	
Parental education level, N (%)							<0.008
Illiteracy	5434(78.5)	1614(76.2)	1857(78.5)	1147(80.0)	551(81.9)	265(80.1)	
Primary school or above	1487(21.5)	505(23.8)	508(21.5)	286(20.0)	122(18.1)	66(19.9)	
Education level, N (%)							<0.001
No formal education	2962(42.8)	790(37.3)	1003(42.4)	654(45.6)	332(49.3)	183(55.3)	
Elementary school	1577(22.8)	493(23.3)	548(23.2)	328(22.9)	150(22.3)	58(17.5)	
Middle school	1573(22.7)	540(25.5)	539(22.8)	307(21.4)	126(18.7)	61(18.4)	
High school and above	809(11.7)	296(14.0)	275(11.6)	144(10.0)	65(9.7)	29(8.8)	
Employment status N (%)							0.298
Agricultural employed	3185(46.0)	980(46.2)	1085(45.9)	643(44.9)	333(49.5)	144(43.5)	
Non-agricultural employed	1694(24.5)	501(23.6)	577(24.4)	381(26.6)	154(22.9)	81(24.5)	
Retired	1916(27.7)	597(28.2)	652(27.6)	390(27.2)	179(26.6)	98(29.6)	
Unemployed	126(1.8)	41(1.9)	51(2.2)	19(1.3)	7(1.0)	8(2.4)	
Drinking status, N (%)							<0.001
Never drink	4071(58.8)	1336(63.0)	1394(58.9)	803(56.0)	350(52.0)	188(56.8)	
Abstainer	543(7.8)	142(6.7)	180(7.6)	118(8.2)	67(10.0)	36(10.9)	
Current drinker	2307(33.3)	641(30.3)	791(33.4)	512(35.7)	256(38.0)	107(32.3)	
Smoking status, N (%)							<0.001
Never smoke	4270(61.7)	1379(65.1)	1451(61.4)	846(59.0)	387(57.5)	207(62.5)	
Former smoker	555(8.0)	157(7.4)	210(8.9)	101(7.0)	60(8.9)	27(8.2)	
Current smoker	2096(30.3)	583(27.5)	704(29.8)	486(33.9)	226(33.6)	97(29.3)	
Digestive diseases, N (%)							<0.001
Absence	5244(75.8)	1692(79.8)	1813(76.7)	1061(74.0)	464(68.9)	214(64.7)	
Presence	1677(24.2)	427(20.2)	552(23.3)	372(26.0)	209(31.1)	117(35.3)	
Respiratory diseases, N (%)							<0.001
Absence	6233(90.1)	1952(92.1)	2150(90.9)	1283(89.5)	581(86.3)	267(80.7)	
Presence	688(9.9)	167(7.9)	215(9.1)	150(10.5)	92(13.7)	64(19.3)	
Arthritis or rheumatism, N (%)							<0.001
Absence	4585(66.2)	1504(71.0)	1607(67.9)	898(62.7)	400(59.4)	176(53.2)	
Presence	2336(33.8)	615(29.0)	758(32.1)	535(37.3)	273(40.6)	155(46.8)	
Cardio-metabolic diseases, N (%)							0.849
Absence	4400(63.6)	1334(63.0)	1502(63.5)	923(64.4)	435(64.6)	206(62.2)	
Presence	2521(36.4)	785(37.0)	863(36.5)	510(35.6)	238(35.4)	125(37.8)	

Notes: ACEs, adverse childhood experiences; N, number; SD, standard deviation. *p* value determined using $\chi 2$ test or analysis of variance F-test.

Table S4. Fit statistics for the trajectories of depressive symptoms after adjusting for gender and baseline age.

Fit statistics	Number of classes				
	**1**	**2**	**3**	**4**	**5**
AIC	-87298.5	-83897.5	-83114.6	-83008.4	-82792.2
BIC	-87315.0	-83938.6	-83180.4	-83094.8	-82907.4
Class, proportion	Class 1, 100%	Class 1, 71.3%	Class 1, 53.6%	Class 1, 27.4%	Class 1, 29.6%
		Class 2, 28.7%	Class 2, 37.1%	Class 2, 42.4%	Class 2, 41.6%
			Class 3, 9.3%	Class 3, 23.8%	Class 3, 11.7%
				Class 4, 6.3%	Class 4, 10.5%
					Class 5, 6.6%
AvePP		Class 1, 0.96	Class 1, 0.91	Class 1, 0.78	Class 1, 0.81
		Class 2, 0.90	Class 2, 0.85	Class 2, 0.73	Class 2, 0.74
			Class 3, 0.89	Class 3, 0.80	Class 3, 0.63
				Class 4, 0.88	Class 4, 0.69
					Class 5, 0.87

Notes: A lower absolute value of AIC and BIC suggests a better model fit. The proportion of each class no less than 5%. A higher value of AvePP is better (preferably > 0.7 in each class). AIC, Akaike information criterion; BIC, the Bayesian information criterion; AvePP, the average posterior probability.

Table S5. Association between ACEs scores and depressive symptoms trajectories by multinominal logistic regression after excluding participants who had memory-related diseases at the 2011 baseline (N = 6850).

**Variables**	**Continuing-low-to-middle vs. Continuing low**		**Continuing-middle-to-high vs. Continuing low**		**Continuing-high vs. Continuing low**	
	**Model 1**	**Model 2**	**Model 1**	**Model 2**	**Model 1**	**Model 2**
	**OR(95%CI)**	**OR(95%CI)**	**OR(95%CI)**	**OR(95%CI)**	**OR(95%CI)**	**OR(95%CI)**
**ACEs scores**						
1	1.241(1.075, 1.434)	1.227(1.061, 1.420)	1.478(1.236, 1.768)	1.434(1.193, 1.724)	2.043(1.459, 2.862)	1.932(1.369, 2.727)
2	1.433(1.206, 1.703)	1.401(1.176, 1.669)	2.048(1.665, 2.519)	1.910(1.543, 2.363)	4.999(3.548, 7.045)	4.363(3.065, 6.210)
3	1.927(1.507, 2.463)	1.863(1.452, 2.389)	3.869(2.940, 5.091)	3.478(2.620, 4.617)	7.717(5.061, 11.767)	6.166(3.987, 9.537)
≥4	2.358(1.598, 3.481)	2.109(1.422, 3.127)	7.363(4.931, 10.996)	5.948(3.942, 8.975)	20.579(12.250, 34.572)	14.611(8.551, 24.967)
**Chronic diseases**						
Digestive diseases		1.496(1.270, 1.763)		2.415(2.012, 2.898)		3.765(2.915, 4.863)
Respiratory diseases		1.493(1.178, 1.892)		1.892(1.448, 2.472)		3.467(2.462, 4.882)
Arthritis or rheumatism		1.745(1.507, 2.022)		2.770(2.346, 3.270)		4.305(3.355, 5.523)
Cardio-metabolic diseases		1.287(1.125, 1.472)		1.488(1.268, 1.746)		1.453(1.137, 1.858)

Notes: The reference group of the multinomial logistic regression model was the continuing-low group. Model 1 was adjusted for gender, age, education level, marital status, hukou status, residence, parental education level, participants' education level, participants' employment status, smoking, and drinking in 2011 baseline survey. Model 2 was additionally included the mediators. ACEs, adverse childhood experiences; OR, odds ratio; CI, confidence interval.

Table S6. KHB mediation analysis of different chronic diseases on the association between ACEs scores and depressive symptoms trajectories after excluding participants who had memory-related diseases at the 2011 baseline (N = 6850).

	**Continuing-low-to-middle vs. Continuing low**				**Continuing-middle-to-high vs. Continuing low**				**Continuing-high vs. Continuing low**			
	**1**	**2**	**3**	**≥4**	**1**	**2**	**3**	**≥4**	**1**	**2**	**3**	**≥4**
**ACEs score**	**OR (95%CI)**	**OR (95%CI)**	**OR (95%CI)**	**OR (95%CI)**	**OR (95%CI)**	**OR (95%CI)**	**OR (95%CI)**	**OR (95%CI)**	**OR (95%CI)**	**OR (95%CI)**	**OR (95%CI)**	**OR (95%CI)**
Total effect	1.264(1.092, 1.462)	1.508(1.265, 1.796)	2.102(1.638, 2.697)	2.557(1.724, 3.792)	1.520(1.264, 1.827)	2.197(1.775, 2.720)	4.390(3.305, 5.831)	8.558(5.667, 12.922)	2.110(1.495, 2.979)	5.398(3.792, 7.684)	8.836(5.714, 13.666)	25.707(15.031, 43.965)
Direct effect	1.227(1.061, 1.420)	1.401(1.176, 1.669)	1.863(1.452, 2.389)	2.109(1.422, 3.127)	1.434(1.193, 1.724)	1.910(1.543, 2.363)	3.478(2.620, 4.617)	5.948(3.942, 8.975)	1.932(1.369, 2.727)	4.363(3.065, 6.210)	6.166(3.987, 9.537)	14.611(8.551, 24.967)
Indirect effect	1.030(0.958, 1.107)	1.076(1.000, 1.158)	1.128(1.046, 1.218)	1.213(1.117, 1.316)	1.060(0.926, 1.212)	1.151(1.005, 1.317)	1.262(1.100, 1.448)	1.439(1.249, 1.657)	1.092(0.894, 1.334)	1.237(1.012, 1.513)	1.433(1.169, 1.757)	1.759(1.428, 2.168)
**Mediators**	**Mediation (%)**				**Mediation (%)**				**Mediation (%)**			
Total indirect effect	12.51	17.82	16.27	20.53	13.86	17.82	15.74	16.94	11.82	12.62	16.51	17.40
Digestive diseases	5.76	5.58	5.99	6.38	7.05	6.36	6.58	6.11	5.94	4.47	6.72	6.07
Arthritis or rheumatism	6.07	10.50	7.90	9.55	6.21	10.01	7.26	7.64	4.99	6.70	7.06	7.24
Respiratory diseases	N.A.	N.A.	2.78	4.53	N.A.	1.74	2.22	3.15	N.A.	1.58	2.94	4.06
Cardio-metabolic diseases	N.A.	N.A.	N.A.	N.A.	N.A.	N.A.	N.A.	N.A.	N.A.	N.A.	N.A.	N.A.

Notes: Model 1 was adjusted for gender, age, education level, marital status, hukou status, residence, parental education level, participants' education level, participants' employment status, smoking, and drinking in 2011 baseline survey. Model 2 was additionally included the mediators. ACEs, adverse childhood experiences; OR, odds ratio; CI, confidence interval; N.A., not applicable.

Table S7. Association between number of ACEs and depressive symptoms trajectories by multinominal logistic regression.

Variables	Continuing-low-to-middle vs. Continuing low		Continuing-middle-to-high vs. Continuing low		Continuing-high vs. Continuing low	
	Model 1	Model 2	Model 1	Model 2	Model 1	Model 2
	OR(95%CI)	OR(95%CI)	OR(95%CI)	OR(95%CI)	OR(95%CI)	OR(95%CI)
**Number of ACEs**	1.230(1.160, 1.304)	1.210(1.140, 1.284)	1.582(1.480, 1.691)	1.515(1.414, 1.623)	2.043(1.858, 2.247)	1.888(1.710, 2.083)
**Chronic diseases**						
Digestive diseases		1.500(1.274, 1.766)		2.425(2.022, 2.908)		3.755(2.917, 4.833)
Respiratory diseases		1.496(1.181, 1.895)		1.939(1.486, 2.530)		3.485(2.488, 4.882)
Arthritis or rheumatism		1.758(1.519, 2.036)		2.793(2.367, 3.296)		4.254(3.327, 5.439)
Cardio-metabolic diseases		1.292(1.130, 1.478)		1.486(1.267, 1.742)		1.554(1.221, 1.978)

Notes: The reference group of the multinomial logistic regression model was the continuing-low group. Model 1 was adjusted for gender, age, education level, marital status, hukou status, residence, parental education level, participants' education level, participants' employment status, smoking, and drinking in 2011 baseline survey. Model 2 was additionally included the mediators. ACEs, adverse childhood experiences; OR, odds ratio; CI, confidence interval.

Table S8. KHB mediation analysis of different chronic diseases on the association between number of ACEs and depressive symptoms trajectories.

	Continuing-low-to-middle vs. Continuing low	Continuing-middle-to-high vs. Continuing low	Continuing-high vs. Continuing low
	**OR (95%CI)**	**OR (95%CI)**	**OR (95%CI)**
Total effect	1.266(1.192, 1.343)	1.651(1.541, 1.769)	2.151(1.948, 2.374)
Direct effect	1.210(1.140, 1.284)	1.515(1.414, 1.623)	1.888(1.710, 2.083)
Indirect effect	1.046(1.033, 1.059)	1.090(1.069, 1.111)	1.139(1.108, 1.172)
**Mediators**	**Mediation (%)**	**Mediation (%)**	**Mediation (%)**
Total indirect effect	19.14	17.15	17.04
Digestive diseases	6.16	6.32	6.19
Arthritis or rheumatism	9.69	8.29	7.65
Respiratory diseases	3.45	2.66	3.29
Cardio-metabolic diseases	N.A.	N.A.	N.A.

Notes: Model 1 was adjusted for gender, age, education level, marital status, hukou status, residence, parental education level, participants' education level, participants' employment status, smoking, and drinking in 2011 baseline survey. Model 2 was additionally included the mediators. ACEs, adverse childhood experiences; OR, odds ratio; CI, confidence interval; N.A., not applicable.

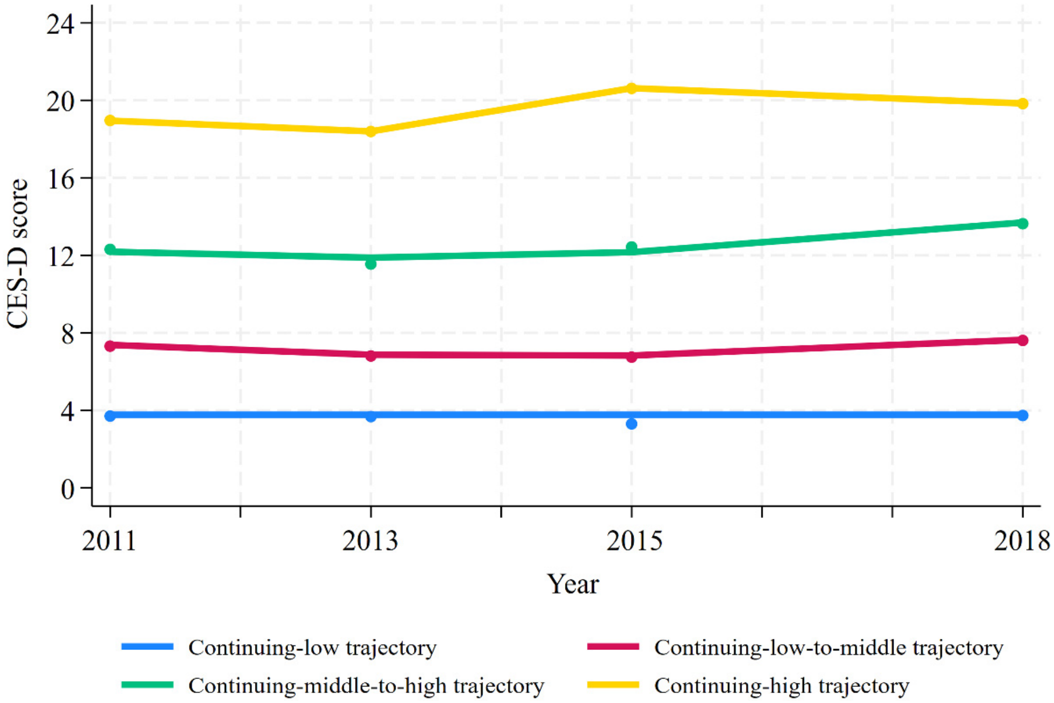

Figure S1. The four distinguished trajectories of depressive symptoms.
